# Amniotic membrane promotes focal adhesion remodeling to stimulate cell migration

**DOI:** 10.1038/s41598-017-15509-z

**Published:** 2017-11-10

**Authors:** Ángel Bernabé-García, Sergio Liarte, Jose M. Moraleda, Gregorio Castellanos, Francisco J. Nicolás

**Affiliations:** 1 Laboratorio de Oncología Molecular y TGF-ß, IMIB-Arrixaca, El Palmar, Murcia, Spain; 2 Unidad de Trasplante y Terapia Celular, Servicio Hematología, Hospital Clínico Universitario Virgen de la Arrixaca, Universidad de Murcia, Murcia, Spain; 3 Servicio de Cirugía General y del Aparato Digestivo, Hospital Clínico Universitario Virgen de la Arrixaca, El Palmar, Murcia, Spain

## Abstract

During wound healing, the migration of keratinocytes onto newly restored extracellular matrix aims to reestablish continuity of the epidermis. The application of amniotic membrane (AM) to chronic, deep traumatic, non-healing wounds has proven successful at stimulating re-epithelialization. When applied on epithelial cell cultures, AM activates extracellular signal-regulated kinases 1/2 (ERK1/2) and c-Jun N-terminal kinases 1/2 (JNK1/2), with the overexpression and phosphorylation of c-Jun along the wound edge. The effect of AM on the migration of cells was investigated by studying critical proteins involved in the focal adhesions turn-over: Focal Adhesion Kinase (FAK), Paxillin and Vinculin. In Mv1Lu and HaCaT cells, validated models for cell migration and wound healing, AM affected the expression and activation of Paxillin, but did not affect Vinculin expression, both factors which integrate into focal adhesions. Moreover, AM regulation also affected FAK activity through phosphorylation. Finally, we have determined that AM regulation of focal adhesions involves both JNK and MEK MAP kinase signaling pathways. This data provides a molecular background to understand how AM regulates critical cell and molecular aspects of cell migration, organizing and directing the movement of cells by the continuous formation, maturation, and turnover of focal adhesion structures at the migration leading edge.

## Introduction

During wound healing, skin integrity is restored by the actions of numerous cell types. Upon skin disruption, molecular events culminate in the formation of a fibrin clot which stops bleeding, while also serving as a provisional matrix for inflammatory cell migration. These are attracted by cytokines, including TGF-ß and other factors released by damaged tissue and platelets^1^. At the end of wound healing, the migration of keratinocytes onto the restored extracellular matrix, which accumulates around the newly formed granulation tissue, culminates this process and so reestablishes the continuity of the epidermis^2^. For the case of deep-traumatic and chronic non-healing wounds, the application of amniotic membrane (AM) has proven to successfully encourage re-epithelialization, resulting in a suitable therapeutic alternative to avoid autologous skin transplantation^3,4^. These properties derive from the ability of the AM to provide immuno-modulatory effects^5^ and to supply helpful growth factors including EGF or TGF-ß, among others^6–8^. In our lab we have shown how, when applied *in vitro*, the presence of AM promotes the activation of R-Smads^8^ and affects the expression profile of several TGF-ß dependent genes in epithelial cultured-cells^9,10^. Moreover, AM induces the activation of extracellular signal-regulated kinases 1/2 (ERK1/2) and c-Jun N-terminal kinases 1/2 (JNK1/2), along with the overexpression of c-Jun on the wound edge^3,8,9^. On top of that, the presence of AM is able to induce the phosphorylation of c-Jun specifically at the migrating front edge of Mv1Lu and HaCaT cells on wound healing assays^8^. In this context, the activation of JNK and MEK signaling, at the edge of healing wounds, is required for the phosphorylation of c-Jun^8^.

Cell migration is a coordinated process that requires the organized and directional movement of cell bodies. This is achieved by rapid changes in the dynamics of actin filaments together with the formation and disassembly of cell adhesion sites^11,12^. The continuous formation of focal complexes (FCs) that mature into focal adhesions (FAs), both containing ready-to-attach proteins^13^, is observed at the actively migrating leading edge of epithelia^14^. Together with protrusion extension and cell body retraction, the adhesion to substrates via the attachment of specific proteins is considered an essential step for cell migration^15^. FCs assembled at cell front undergo either rapid turnover or maturation in response to contractile forces and the feedback from extracellular components^13,14^. Many proteins are involved in the assembly, maturation and disassembly of FAs^11,16^. Among them, Paxillin and Vinculin can be considered fundamental components for FAs functioning, as once incorporated, the first is able to integrate multiple signaling cascades to coordinate the process^16^, while the latter has a broader role related to cytoskeletal dynamics^16^. Moreover, Paxillin acts as a key scaffold protein in the formation of FAs, whose assembly and disassembly is so critical during cellular migration^17^. On top of these proteins, the Focal Adhesion Kinase (FAK) acts as a master regulator^11^ of the process since it can modulate FAs assembly and disassembly through phosphorylation of different players, including Paxillin^13^.

In this paper, we applied the wound scratch assay method along with specific inhibitors for MEK-1 and JNK signaling pathways to study *in situ* the effect of AM on focal structures, either FCs or FAs, dynamism during the migration of two well established migration and wound healing cellular models, the Mink Lung Epithelial (Mv1Lu) cells and the spontaneously immortalized human keratinocyte (HaCaT) cells. By paying attention to focal structures and the proteins involved in this biological phenomenon, we describe the ability of AM treatment to stimulate the dynamics and turnover of focal structures contributing to enhanced migration of cells at the front edge of wounds.

## Results

### AM treatment induces the expression of Paxillin in Mv1Lu cells

**Figure 1:**
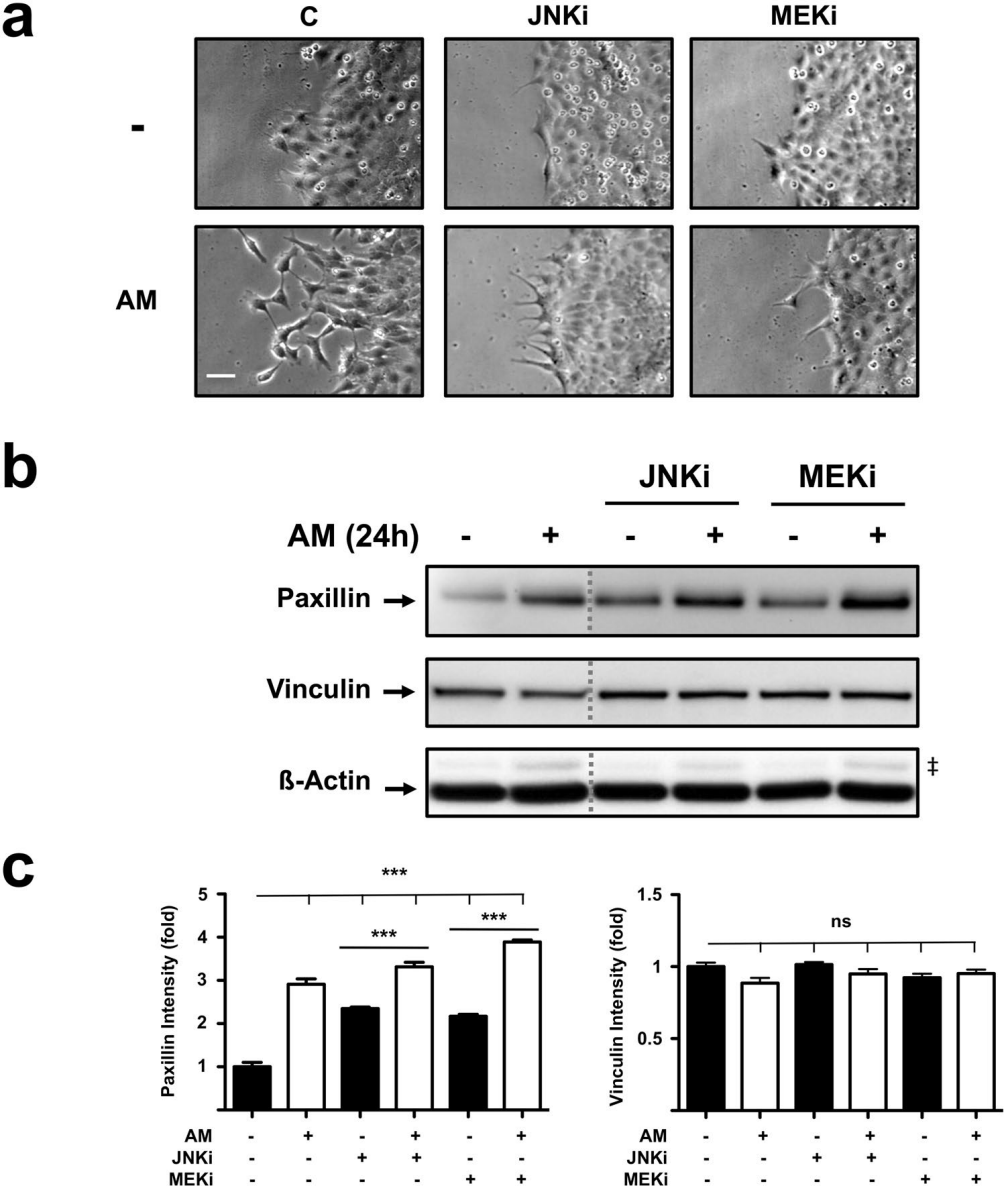
Amniotic membrane (AM) promotes cell protrusion generation and Paxillin expression in migrating Mv1Lu cells. (**a)** Detailed pictures of the migrating edge of artificial wound assays treated with AM in combination with inhibitors. Scale Bar 50 µm. (**b)** Western Blot of total protein extracts from sub-confluent Mv1Lu cells cultured in the presence of AM and/or inhibitors and collected after 24 hours. The dashed grey lines indicate that two distant parts of the very same blot were put together. ß-actin was used as loading control. (‡) Unspecific bands. (**c)** Relative protein level plots generated from Western Blot quantification. C: serum starvation; JNKi: SP600125; MEKi: PD98059. Asterisks denote statistically significant differences between conditions according to ANOVA statistical analysis: (***) p < 0.001; (ns) not significant. All experiments were repeated at least three times. Representative pictures and results are shown.

Non-malignant mink lung epithelial cells, Mv1Lu, are recognized as a good epithelial model for the study of cell motility due to their ability to migrate and stop proliferation by means of cell-to-cell contact inhibition after confluence^18–21^. A closer view of the migration edge of artificial wound assays performed on Mv1Lu cells^8,9^ revealed the ability of AM to stimulate protrusion generation at the migrating edge of wounds (Fig. 1a). The presence of SP600125 or PD98059, inhibitors for JNK and MEK (JNKi and MEKi) respectively, produced an apparent decrease in the number of protrusions at the edge of migrating cells (Fig. 1a). These variations in the number of protrusions accompany variations in cell motility and are regarded as an indicator for increased cytoskeletal dynamics, in which Paxillin is usually involved^14^. When we studied Paxillin expression by performing WB on total protein extracts obtained from sub-confluent Mv1Lu cells, so that they would behave like a migratory edge^8^, we detected that AM treatment resulted in increased Paxillin levels (Fig. 1b and c). Paxillin activities are regulated through posttranslational modification, including phosphorylation and ubiquitination^22^. JNK and MEK MAP kinases had been shown to take part in the mediation of some of the effects triggered by AM stimulation^8,9^. The presence of either JNKi or MEKi promoted the accumulation of Paxillin after 24 hours in Mv1Lu sub-confluent cells. Interestingly, neither JNKi nor MEKi presence affected Paxillin induction by AM stimulus, which promoted its accumulation in a similar fashion independently of the presence of inhibitors (Fig. 1b and c). In this line, we also studied Vinculin total levels, as a concurrent factor incorporating to FAs. In that case, the expression levels of Vinculin showed not to be affected either by AM stimulus or by the presence of JNKi or MEKi (Fig. 1b and c).

These results would suggest the ability of AM treatment to promote cytoskeletal changes specifically involving the modulation of Paxillin protein levels in Mv1Lu cells. Moreover, our results would indicate that within the context of AM stimulation, this modulation would be influenced by MAP kinase activities.

### Increased Paxillin levels correspond with enhanced focal complex dynamics in migrating Mv1Lu cells

**Figure 2:**
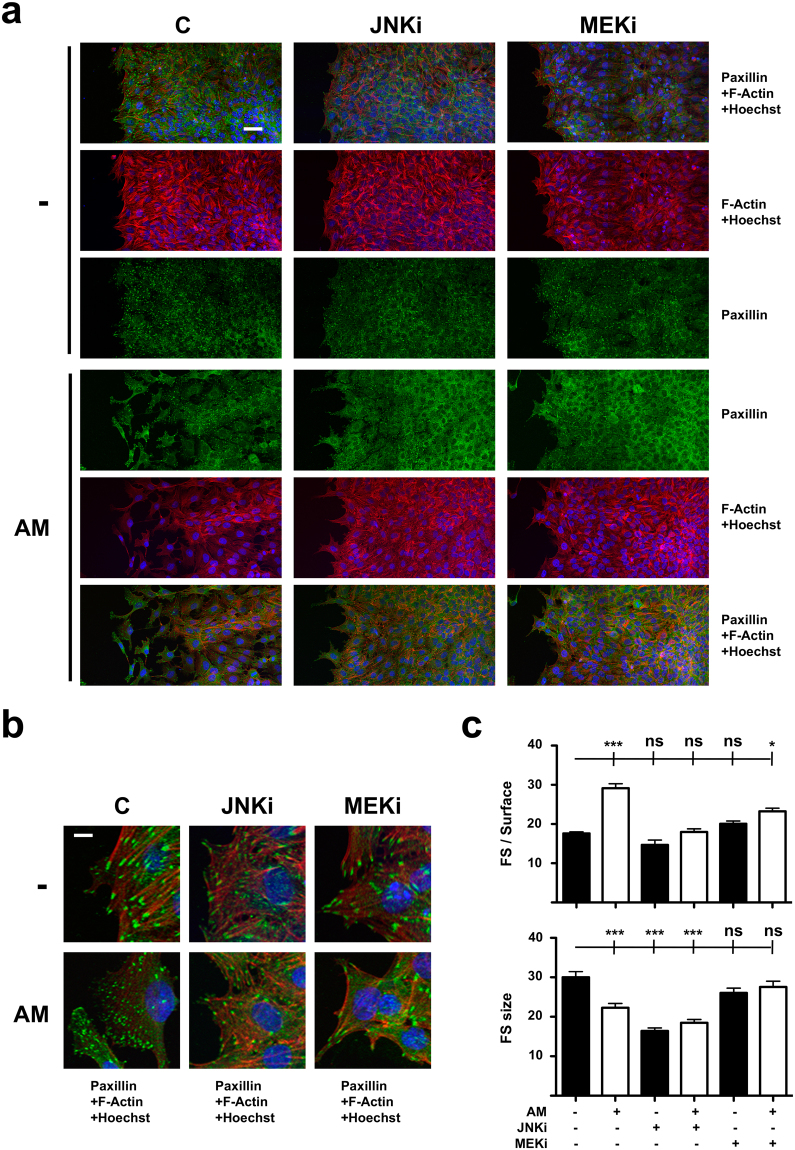
Amniotic membrane (AM) treatment alters Paxillin distribution and focal structures arrangement in migrating Mv1Lu cells. (**a)** Confocal microscopy images of Mv1Lu cells at the migrating edge of artificial wound assays. Scale Bar 50 µm. Juxtaposed field images were taken to show migration front from a wider perspective. (**b)** 2.5x magnified detail from merged pictures in (**a**). Scale Bar 10 µm. (**c)** Plots for average number and size of focal structures detected at the migrating leading edge. Asterisks denote statistically significant differences between conditions according to ANOVA statistical analysis: (*) p < 0.05, (**) p < 0.005 and (***) p < 0.001; (ns) not significant. Paxillin: green; Actin: red; Nuclei: blue; C: serum starvation; JNKi: SP600125; MEKi: PD98059; FS: focal structures. All the experiments were repeated at least three times. Representative pictures are shown.

When studying cells at the leading edge of wound assays by means of confocal microscopy, the cells migratory status could be determined by paying attention to the conformation of actin filaments: actively migrating cells show a rather diffuse structure; less mobile cells usually depict a profuse filament network^23^ (Fig. 2a). In our case, immuno-labelling of Paxillin on Mv1Lu cells also revealed distinct patterns for this protein. While unstimulated cells showed well defined focal structures corresponding to consolidated FAs, AM-stimulated cells depicted abundant but less conspicuous labelling at the migrating membrane, probably corresponding with the conformation of FCs (Fig. 2a). Both JNKi and MEKi are known to hamper AM induction of migration and wound closure in scratch assays^8,9^. When we studied the effects of these inhibitors on the cytoskeletal organization of unstimulated Mv1Lu cells no alteration was observed. However, in the case of AM-treatment, the cells lining the wound gap showed a consistent filament network in the presence of inhibitors, contrasting with the diffuse cytoskeleton labelling observed in their absence (Fig. 2a). For the case of Paxillin immuno-labelling, clear changes were observable upon AM stimulation in the presence of inhibitors (Fig. 2a and b). It is worth to note how AM stimulation alone increased the number of focal structures detected at the leading edge of migrating fronts. However, the presence of both JNKi and MEKi inhibitors when AM was added, restrained the increase of observable focal structures at that area (Fig. 2c). Interestingly, both AM stimulation or the presence of JNKi resulted in a reduction of the average size of the detected focal structures, while MEKi did not reduce significantly this parameter (Fig. 2c).

Altogether, these observations would corroborate the ability of AM to promote the migration of Mv1Lu cells by encouraging the integration of Paxillin into focal structures, also involving changes to the conformation of actin filaments. Moreover, our results suggest that this phenomenon would be overwatched by MAP kinase activities.

### AM treatment promotes Paxillin activation in migrating HaCaT cells

**Figure 3:**
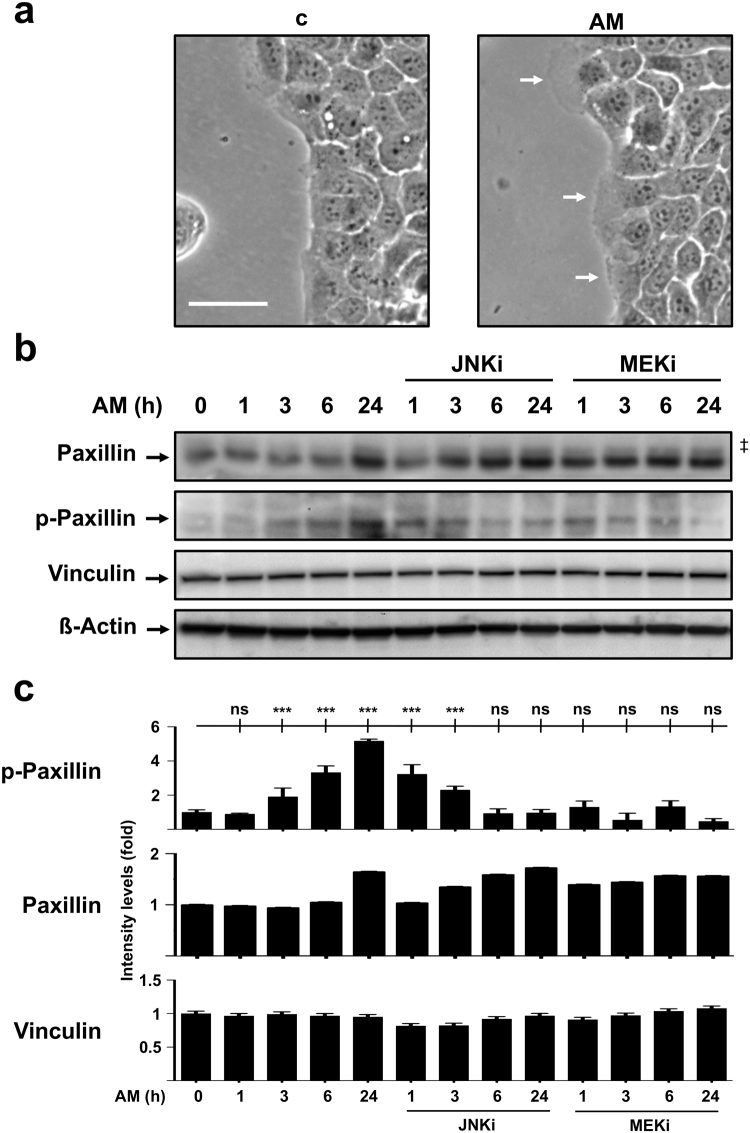
Amniotic membrane (AM) promotes cell protrusion generation and Paxillin phosphorilation in migrating HaCaT cells. (**a)** Migrating HaCaT cells, protrusions are indicated by arrows. Scale Bar 50 µm. (**b)** Western Blot of total protein extracts from sub-confluent HaCaT cells cultured in the presence of AM and/or inhibitors and collected after 24 hours. ß-actin was used as loading control. (‡) Unspecific bands. (**c)** Relative protein level plots generated from Western Blot quantification data. C: serum starvation; JNKi: SP600125; MEKi: PD98059. Asterisks denote statistically significant differences between conditions according to ANOVA statistical analysis: (***) p < 0.001; (ns) not significant. All experiments were repeated at least three times. Representative pictures and results are shown.

Spontaneously immortalized human keratinocyte cells, HaCaT, are highly valued as a human skin and epithelial wound healing model. HaCaT cells have a non-malignant origin and exhibit the ability to stop proliferation upon cell-to-cell contact inhibition. Finally, they also retain normal keratinization and migratory capacities upon confluence disruption^8,9,24^, also showing a considerable response in terms of wound closure to AM treatment^8,9^. A closer look at the migrating leading edge of sub-confluent HaCaT cells also revealed increased extension in protrusions of cells exposed to AM (Fig. 3a). When we studied Paxillin expression by WB, we detected that AM treatment resulted in an increase of Paxillin levels, which accumulated considerably after 24 hours (Fig. 3b and c). Interestingly, the presence of either JNKi or MEKi alone increased Paxillin levels (data not shown), nevertheless AM stimulation along with inhibitors resulted in anticipated augmented Paxillin levels (Fig. 3b and c). To further understand the underlying mechanism, we tried to assess Paxillin activation status by researching into its phosphorylated form. Phospho-Paxillin levels increased steadily upon AM treatment, showing its highest at the end of the 24-hour period. Interestingly, while the presence of JNKi initially promoted a substantial activation of Paxillin up to 3 hours, the presence of either JNKi or MEKi hampered the ability of AM to promote such activation at longer incubation times (Fig. 3b and c). Lastly, in a similar fashion to Mv1Lu cells, total expression levels of Vinculin remained steady, independently of either AM stimulation or JNKi and MEKi co-treatment.

These results suggest that upon AM stimulation, similar mechanisms would operate both in HaCaT and Mv1Lu cells for the promotion of the cytoskeletal changes required for cell migration and wound restoration.

### AM induced Paxillin dynamics are associated with cytoskeletal filament conformation in migrating HaCaT cells

**Figure 4:**
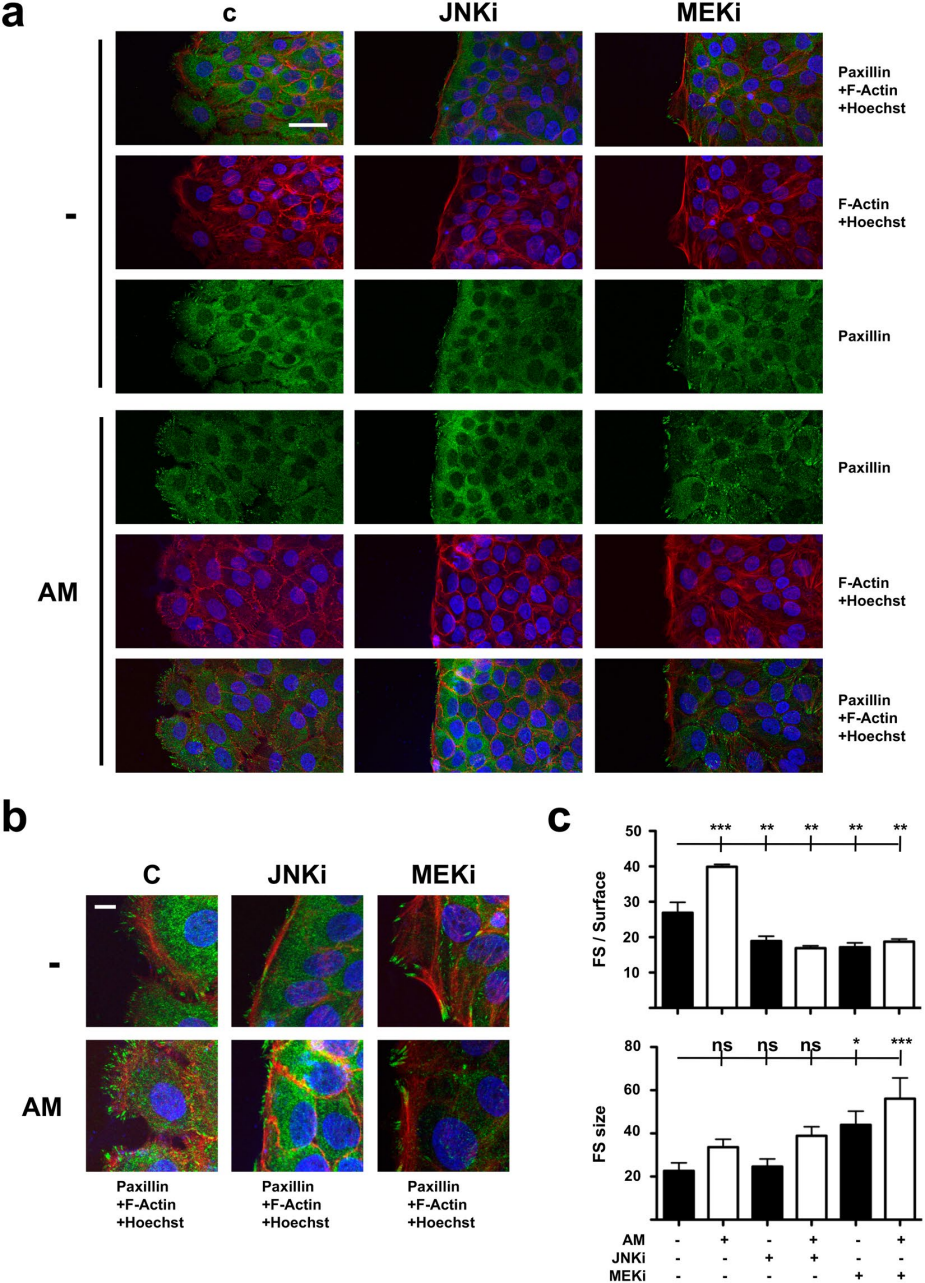
Amniotic membrane (AM) treatment increases the detection focal structures in migrating HaCaT cells. (**a)** Confocal microscopy images of HaCaT cells at the migrating edge of artificial wound assays. Scale Bar 50 µm. (**b)** 2.5x magnified detail from merged pictures in (a). (**c)** Plots for average number and size of focal structures detected at the migrating leading edge. Asterisks denote statistically significant differences between conditions according to ANOVA statistical analysis: (*) p < 0.05, (**) p < 0.005 and (***) p < 0.001; (ns) not significant. Paxillin: green; Actin: red; Nuclei: blue; C: serum starvation; JNKi: SP600125; MEKi: PD98059; FS: focal structures. All the experiments were repeated at least three times. Representative pictures are shown.

Confocal microscopy studies of artificial wounds on HaCaT cells showed a highly coherent monolayer an orderly cell disposition at the wound edge. Interestingly, when studying actin filaments, we found that cells treated with either JNKi or MEKi, regardless of AM presence or not, showed intense labelling corresponding with a well-developed filament network at the protruding cellular pole of cells bordering the wound gap. In contrast, control or AM-treated cells portrayed weaker labelling intensity at the migrating edge compared with areas distant from the wound gap (Fig. 4). When focusing on Paxillin immuno-labelling, HaCaT cells showed a diffuse pattern, in which focal structures were mainly appreciable at the edge of the migrating front (Fig. 4a and b). Interestingly, the treatment with AM promoted an increase in the number of detectable focal structures, which was abrogated by the presence of any of the inhibitors used (Fig. 4c). However, in contrast to what was observed for Mv1Lu cells, the stimulation of wounded HaCaT cells with AM did not result in a significant increase of the focal structures average size of at the leading edge of the migrating front (Fig. 4c). Moreover, while JNKi presence seemingly had no effect on the evolution of the average size of these focal structures, the presence of MEKi promoted a noticeable increase on it, which was further contributed by AM stimulus (Fig. 4c).

Altogether, these observations would sustain the ability of AM treatment to promote Paxillin dynamism and cytoskeletal remodeling for the promotion of HaCaT cells migration.

### MA-induced Paxillin dynamics correlate with increased Focal Adhesion Kinase activation at the migrating edge of HaCaT cells

**Figure 5:**
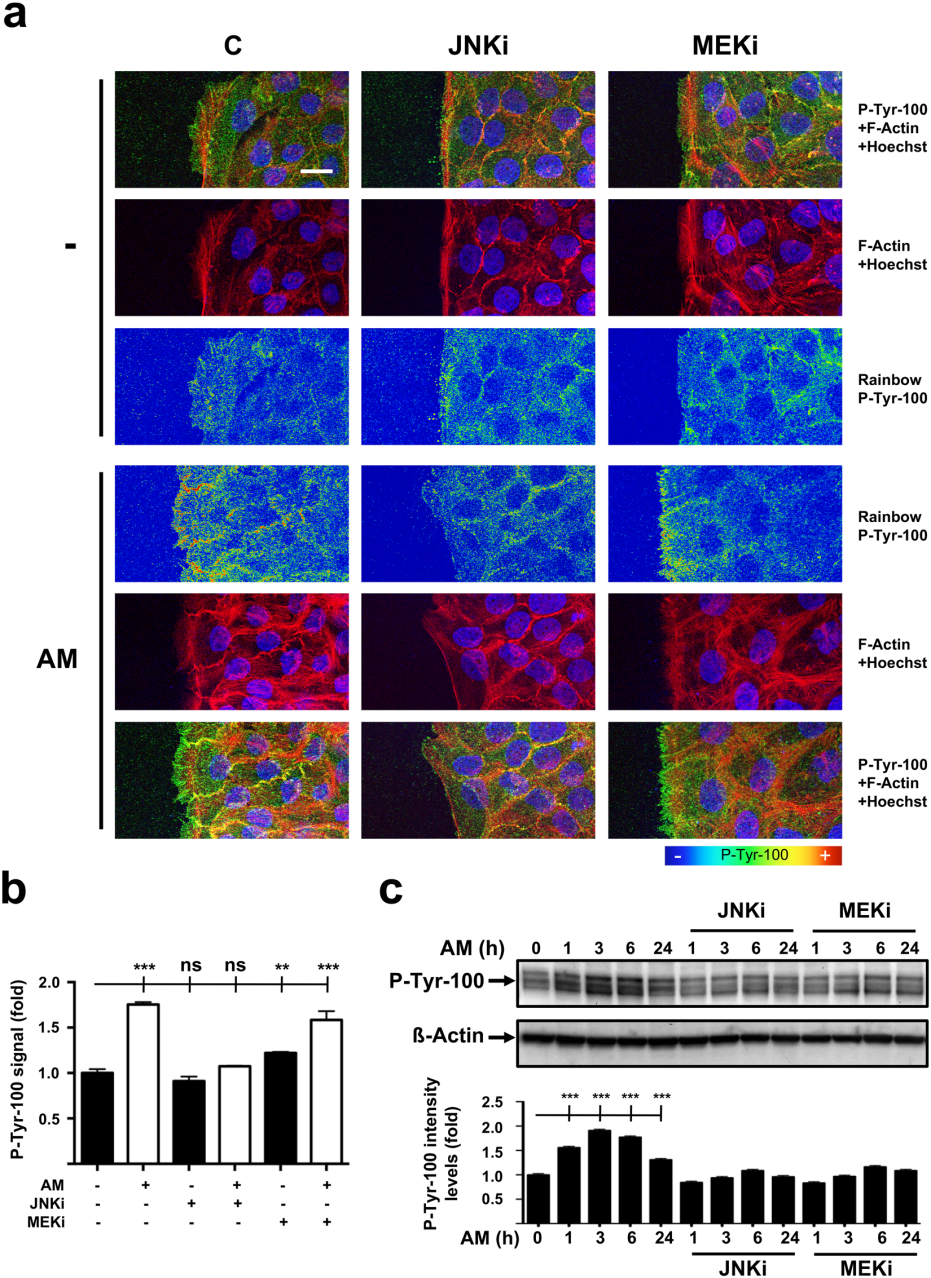
Amniotic membrane (AM) promotes FAK activation and detection at focal structures in migrating HaCaT cells. (**a**) HaCaT cells at the migrating edge of artificial wound assays show increased p-tyr-100 immune-staining when AM was present. P-tyr-100 immune-staining signal was converted into pseudo-color rainbow scale to display fluorescence-intensity variations. Fluorescent signal was converted using Rainbow feature from ZEN software, on a linear mode and covering the full range of the data. Co-staining with phalloidin and Hoechst-33258 was used to show the cell structure and nuclei, respectively. P-tyr-100: pseudo-color; p-tyr-100: green; Actin: red; Nuclei: blue. Scale Bar 25 µm. (**b)** Relative fluorescence level plot generated from average p-tyr-100 signal intensity data obtained at the migrating leading edge. (**c)** Western Blot of total protein extracts from sub-confluent HaCaT cells cultured in the presence of AM and/or different inhibitors collected for a 24 hours time course. ß-actin was used a loading control. Relative protein level plots were generated from Western Blot quantification data. C: serum starvation; JNKi: SP600125; MEKi: PD98059. Asterisks denote statistically significant differences between conditions according to ANOVA statistical analysis: (***) p < 0.001; (ns) not significant. All the experiments were repeated at least three times. Representative pictures and results are shown.

FAK is another relevant factor in modulating focal structures assembly, as well as in cytoskeletal remodeling and in the promotion of directional cell movement. When we studied FAK behavior on cells lining artificial wounds by means of immuno-labelling of phosphorylated tyrosine residues^25^, consolidated staining was detectable on the leading edge of unstimulated cells (Fig. 5a). Interestingly, AM stimulation resulted in increased immuno-labelling of migrating HaCaT cells. It is worth noting, as showed by pseudo-color analysis, that the labelling signal highly condensates at the membrane leading edge, especially in the cases of AM stimulated cells (Fig. 5a and b). Strikingly, immuno-labelling of cells treated with AM and either JNKi or MEKi depicted weaker, although some increase in fluorescence signal was recorded in cells co-exposed to AM and MEKi inhibitor (Fig. 5a and b). To further validate the previous observations, we performed WB on total protein extracts obtained from sub-confluent HaCaT cells and studied phospho-tyrosine detection as an optimal readout for FAK activation^25^. Coherently, AM stimulation resulted in a clear increase in phospho-tyrosine detection, which was sustained for the first 6 hours of treatment, retaining considerable induction after 24 hours (Fig. 5c). Consistently, despite AM inoculation, the presence of JNKi restrained phospho-tyrosine levels similar to those registered for control cells along the 24-hour time course (Fig. 5c). However, when MEKi and AM were used, although an increase in phospho-tyrosine detection was recorded, this time it was much weaker than in samples stimulated just with AM (Fig. 5c).

In conclusion, these data show evidence of the participation of FAK in the dynamism affecting FAs in migrating HaCaT cells after AM stimulation, through a process which involves the modulation of FAK activities by means of its phosphorylation status.

## Discussion

In this paper, using two validated cell models for studying migration and wound healing, we have shown how the ability of AM to promote migration is sustained in the reorganization of cell focal structures, with the particular implication of Paxillin and FAK. AM treatment clearly induced the accumulation of Paxillin in both Mv1Lu and HaCaT cells. In both cases, the effect of AM on Paxillin protein levels was not blocked in the presence of either MEKi or JNKi. When we looked at Vinculin, another protein participating focal structures, AM did not exert relevant regulation on its protein levels. Notably, AM treatment induced the phosphorylation of Paxillin in HaCaT cells; in that case however, the presence of MEKi or JNKi prevented Paxillin phosphorylation. Moreover, AM caused a net remodeling of the FAs clearly noticeable by the alteration in size and number of focal structures detected at the protruding pole of cells at the migrating edge. This re-arrangement was disturbed by the presence of JNKi and MEKi, coherently with their well-known capacity to inhibit AM-induced cell migration^8,9^. Additionally, we have shown the ability of AM treatment to trigger the enrichment of active FAK to the migrating cells protruding pole, a phenomenon that was again affected by the presence of MEKi and JNKi.

In artificial wound assays, a close look at the wound migrating edge of epithelial cells shows evident morphological differences in those cells actively migrating compared with less motile cells. These morphological differences generally correspond with the development of cytoplasmic protrusions such as filopodia and ruffles^14^, which as we show in this paper, increase when cells are stimulated with AM. These differences could be swiftly related to divergent behavior involving focal as well as cytoskeletal structures. Directional cell movement requires the continuous formation of focal complexes (FCs), maturation into focal adhesions (FAs), and turnover at the rear of the leading-edge migrating cells^13,14^. FCs assembled at cell front undergo either rapid turnover or maturation in response to contractile forces and the feedback from extracellular components^13,14^. In this process, Paxillin acts as a key scaffold protein for the assembly, maturation into FAs and disassembly^26^. By providing a structural framework, Paxillin facilitates the concurrent binding of different protein components, particularly signaling molecules. This feeds an efficient crosstalk between different pathways, affecting many fundamental processes including gene transcription^27^. Coherently, alterations in Paxillin expression levels can affect migratory performance, as shown by different classic reports linking its expression with cancer metastatic potential^28–30^. Here, we show how AM treatment increased Paxillin expression in migrating Mv1Lu and HaCaT cells, in a process which was not affected when JNK or MEK-1 signaling was inhibited. Interestingly, although knowledge on the mechanisms affecting Paxillin expression is scarce, it has been noted that TGF-ß1 signaling promotes its accumulation through a transcription-independent mechanism^31^. As TGF-ß1 is considered among the factors released by AM, here levels of Paxillin may be regulated by transcription as well as by protein stabilization in response to this kind of signaling. Vinculin, another important component incorporating to FAs, has the main function of anchoring actin filaments and integrins^16^. Interestingly, and in contrast to Paxillin, Vinculin was not transcriptionally regulated by the presence of AM. Although both proteins co-localize at FAs, they openly diverge in their ability to transduce signals, as the stimuli conducted through Vinculin fundamentally influence cytoskeletal mechanical properties, such as contractibility, adhesion strength and stiffness^16,32^. The ability of the AM treatment to induce this differential expression, further suggests its ability to accurately activate chosen components of the migration machinery.

Activation of JNK is usually correlated with increased cell migration in a mechanism involving the phosphorylation of known substrates. This process results in the transcriptomic modulation of key genes required for migration such as *c-JUN* ^33^. Indeed, on top of the ability of AM to induce JNK signaling^3^, we have described in the past the increased expression of c-Jun^9^ and the occurrence of its phosphorylation specifically at the leading edge of migrating Mv1Lu and HaCaT cells when they were stimulated with AM^8^. However, a more dynamic role for JNK in regulating cell migration involves the direct phosphorylation of cytosolic substrates. Paxillin exhibits several phosphorylation sites whose modifications modulate its behavior, already been identified as a substrate for JNK^34^. In this context, Paxillin interaction with activated JNK seems critical, as the resulting phosphorylation at Ser-178 residue appears to be essential to allow migration^22,35^. As we have shown here, AM treatment resulted in an obvious rise in Paxillin Ser-178 residue phosphorylated levels in HaCaT cells. More importantly, the presence of JNKi not only altered AM-induced Paxillin phosphorylation but also abrogated its incorporation into nascent FCs despite increasing Paxillin levels. Indeed, this was evidenced by the lower number of focal structures detected at the wound’s leading edge cells. It is worth noting that it has been shown that different extracellular signals regulate spreading and cell migration through this JNK/Paxillin system^13^. The fact that such observations were not as dramatic when using MEKi, which merely contributes to JNK activation^8,9^, would further point to activated JNK as a key element channeling the effects of AM on FAs dynamics and HaCaT cell migration.

It has been described that phosphorylation of Paxillin by JNK further promotes its association with FAK^22,35^. FAK operates as the cell master controller for FAs remodeling at the migration leading edge, consequently driving directional cell movement^11,13^. Increased FAK localization at these areas has been connected to FAs increased turnover at the protrusion front^13^. We have observed a clear change in the number and size of focal structures in cells stimulated with AM when compared to unstimulated control cells, both by studying Paxillin and phospho-tyrosine FAK. Paxillin is phosphorylated at residues Tyr-31 and Tyr-118 by FAK^27,35^. Phosphorylation at these sites generates two functional binding elements for members of the Crk family of SH2-SH3 adaptor proteins, which further coordinate integrin-mediated cell motility and consolidation of FCs into FAs^27^. This process is crucial, as mature FAs enable the cell contractibility necessary to pull the cell forward^13^. Additionally, FAK integrates growth-factor and integrin signals to promote cell migration^11,36^. Coherently, FAK-null fibroblasts show larger, more stable focal adhesions and lose random migration capacity^37^. Here we have shown the ability of AM to promote a strong stimulation of FAK, as evidenced by immunostaining at the leading edge. This would agree with the idea of the emergence of numerous dynamic FCs in cells stimulated with AM, compared to those left alone which show a rather static behavior as could be related to the reduced number and size of focal structures. Indeed, in every case, the structure of actin microfilaments was also suggestive for the migratory status of the conditions assayed^14,38^. Although the best characterized activation mechanism for FAK involves its auto-phosphorylation at Tyr-397 induced by interaction with Extra Cellular Matrix (ECM) components and members of the integrin family^11,16^, here we evidenced the ability of both JNKi and MEKi to prevent further FAK activation by AM. Whether this phenomenon is the consequence of higher ECM feedback linked to increased migration, or the result of additional endogenous or exogenous receptor activation by AM, perhaps also involving JNK, is something that will be the object of further research.

Migrating cells require for the spatiotemporal integration of many signals regulating essential activities responsible for cell movement. Continuous assembly/disassembly of cell adhesion sites, together with cytoskeleton dynamics are coordinated by those signals^12^. By regulating critical and intimate mechanisms inherent to these processes, AM is able to consistently stimulate cell migration. Precisely, using two distant but relevant epithelial models for the study of cell migration and wound healing such as Mv1Lu and HaCaT cells, here we showed evidence of the involving of MAP kinases activities for the promotion of FAs remodeling, a fundamental phenomenon for cell migration. Understanding this regulation and its potential implications for different cell types involved may help explain some of the wonderful properties shown by AM for the epithelialization of clinical non-healing wounds, providing a real opportunity for the development of improved therapies and procedures.

## Materials and Methods

This study was approved by the local ethics committee (University Clinical Hospital Virgen de la Arrixaca, Murcia, Spain) and the Spanish Agency for Drugs (AEMPS). Appropriate written informed consent was obtained from AM donors. Additionally, all methods were performed in accordance with the relevant guidelines and regulations.

### Cells and AM processing

Mink Lung Epithelial cells (Mv1Lu)^18,20,21^, a kind gift from Dr. Inman, were grown in Eagle’s Minimum Essential Medium (EMEM) and spontaneously immortalized human keratinocyte (HaCaT) cells, obtained from ATCC, were cultured in Dulbecco’s Modified Eagle Medium (DMEM) high-glucose (Biowest, Nuaillé, France). Both cell lines media were supplemented with 10% Fetal Bovine Serum (FBS) (Thermo Fisher Scientific, Waltham, MA USA), 1000 units/ml Penicillin/1000 µg/ml Streptomycin (Sigma-Aldrich, St Louis, MO, USA), and 1% L-glutamine (Biowest, Nuaillé, France) at 37 °C in a 7.5% CO_2_ incubator. Amniotic membrane was prepared as described elsewhere^9^. Briefly, term placenta from healthy donor mothers was obtained from uncomplicated caesarean section. The fetal membranes were washed in Physiological Saline Solution (PSS) (B.Braun, Barcelona, Spain) supplemented with 50 µg/ml Amphotericin (Bristol-MyersSquibb, Madrid, Spain), 48 µg/ml Clotrymazol (Almirall-Prodesfarma, Barcelona, Spain), 50 µg/ml Tobramycin (Laboratorios Normon, Madrid, Spain) and 50 µg/ml Vancomycin (Laboratorios Hospira, Madrid, Spain) and rapidly transferred to the laboratory in sterile conditions. Under a laminar flow cabinet, the amnion was mechanically peeled from the chorion, washed three to four times with 200 ml of PBS (Biowest, Nuaillé, France) and flattened onto sterile nitrocellulose paper (Pierce, Thermo Fisher Scientific, Waltham, MA USA) with the amniotic epithelium surface up, and the spongy layer facing and sticking to the nitrocellulose paper. Then, paper with adhered membrane was cut into 1 cm × 1 cm fragments. Freshly cut AM fragments were separated from paper pieces and placed in a sterile vial containing a freezing solution made of 10% Dimethyl Sulfoxide (DMSO) (Sigma-Aldrich, St Louis, MO, USA), 4% human albumin (Grifols, Bercelona, Spain) in DMEM (Biowest, Nuaillé, France) medium and then frozen at −80 °C and later preserved in liquid nitrogen until further use. The day of wound application, AM pieces were thawed at 37 °C, then pieces were washed three times with DMEM and placed at 37 °C in a 7.5% CO_2_ incubator for two hours for the re-vitalisation of the amniotic membrane cells. The pieces now were used for the desired experiment. When the JNK inhibitor SP600125 (15 µM; JNKi) or MEK inhibitor PD98059 (50 µM; MEKi) (all Sigma-Aldrich, St Louis, MO, USA) were used, these were added at the beginning (T_0_) alone or with pieces of AM. In all cases, similar concentrations of DMSO (inhibitor’s carrier) or PBS were used for control samples.

### Western blot

Mv1Lu or HaCaT cells were seeded to reach 50% confluence on day two in 6-cm diameter plates using a medium containing 10% FBS. At this point, culture medium was substituted for a FBS-free medium and cells were cultured on it for an additional 24-hour period. The treatment with AM was done as previously described^9^. Briefly, 5 pieces of 1cm^2^ AM per plate were placed floating on the medium of the samples indicated with AM in the figures. At T_0_, incubation with AM, inhibitors alone, or combination of both began. After 24 hours incubation, AM was removed and cells were lysed using lysis buffer: 20 mM TRIS pH 7.5, 150 mM NaCl, 1 mM EDTA, 1.2 mM MgCl2, 0.5% Nonidet P-40, 1 mM DTT, 25 mM NaF and 25 mM ß-glycerolphosphate (all from Sigma-Aldrich, St Louis, MO, USA) supplemented with phosphatase inhibitors (I and II) and protease inhibitors (all from Sigma-Aldrich, St Louis, MO, USA). Protein extracts were analyzed by SDS-PAGE followed by western blot using the appropriate antibodies: anti-Paxillin^39^ (Abcam, Cambridge, UK), anti-phospho-Paxillin^40^ (Life Technologies Corporation, Carlsbad, CA, USA), anti-phospho-tyr-100^25^ (New England Biolabs, Frankfurt, Germany), anti-vinculin^41^ and anti-β-actin^42^ (Sigma-Aldrich, St Louis, MO, USA). Secondary antibodies were anti-rabbit IgG Horseradish peroxidase linked F(ab’)_2_ I fragment (from donkey) (GE Healthcare, GE, Little Chalfont, United Kingdom) and Horseradish peroxidase linked Rat anti-mouse IgG_1_ (BD Pharmingen, Beckton Dickinson, Franklin Lakes, NJ, USA). WBs were developed using Horseradish peroxidase substrate (ECL) (GE Healthcare, GE, Little Chalfont, United Kingdom) and images were taken with a Chemidoc XRS® (Bio-Rad, Hercules, Ca, USA).

### Immunocytochemistry and analysis in wound healing assay

Mv1Lu or HaCaT cells were grown to confluence over round glass-coverslips using medium containing 10% FBS (Thermo Fisher Scientific, Waltham, MA USA). Prior to the assays (24 hours), cells were washed and medium was exchanged for FBS-free medium. Then, the epithelium was wounded using a razor blade, which was immediately dragged to create a space large enough to allow migration. At T_0_, fresh FBS-free medium was placed and incubation began in a 6-well plate containing five coverslips and four 1 cm^2^ piece of AM floating on the medium. When used, inhibitors were added at T_0_. After 24 hours of incubation, AM was removed and the coverslips were fixed in 4% formaldehyde (Applichem GmbH, Darmstadt, Germany) in PBS (Biowest, Nuaillé, France). Subsequently, cells were permeabilized with 0.3% Triton X-100 (Sigma-Aldrich, St Louis, MO, USA) in PBS (Biowest, Nuaillé, France). Immunostaining was performed by blocking buffer [0.3% Bovine Serum Albumin (BSA) (Santa Cruz Biotechnology, Heidelberg, Germany), 10% FBS (Thermo Fisher Scientific, Waltham, MA USA), 0.1% Triton X-100 (Sigma-Aldrich, St Louis, MO, USA) in PBS (Biowest, Nuaillé, France)] supplemented with 5% skimmed milk (Beckton Dickinson, Franklin Lakes, NJ, USA), at room temperature for 1 hour of incubation with either: anti-Paxillin (Abcam, Cambridge, UK) or anti-phospho-tyr-100 (Cell Signaling Technology, Danvers, MA, USA) performed in blocking buffer. After staining with the primary antibody, samples were stained with the appropriate fluorescent-labelled secondary antibodies together with Alexa Fluor 594-conjugated phalloidin (Molecular Probes, Thermo Fisher Scientific, Waltham, MA USA), where indicated, and Hoechst 33258 (Fluka, Biochemika, Sigma-Aldrich, St Louis, MO, USA) for half an hour at room temperature to reveal actin and nuclei respectively. Control samples without primary antibody were done, showing in all cases a negative signal, to test the specificity of secondary antibody (data not shown). Finally, samples were examined and images were taken with a confocal microscope (LSM 510 META from ZEISS, Jena, Germany) using Zeiss Efficient Navigation (ZEN) interface software. For the acquisition of images, a Plan-neofluar 40x/1.30 oil DIC lens was used. Fields were scanned with the appropriate laser and fluorescent signal was collected with the scanning laser confocal module Meta, Zeiss. When a wider view of the migration front was required (indicated in the figure), two juxtaposed fields were acquired utilizing the “Tile scan” feature of ZEN. For clarity of some images, fluorescent signal was converted on a linear mode and covering the full data range using Rainbow feature from ZEN software.

### Quantitative image processing and statistical analysis

Delimitation of regions of interest and quantification of structures was performed using built in tools included in Image J 1.8.0 image processing software. In all cases, we used Prism’s Graph Pad software for data calculation and representation. Statistical analysis shown in figures represent data mean ± SEM, analyzed using one way ANOVA statistical analysis applying Newman-Keuls multiple comparison test. At the figure legends, asterisks denote statistically significant differences between treatments (*p < 0.05, **p < 0.005 and ***p < 0.001, ****p < 0.0001).

### Data availability

All data generated or analyzed during this study are included in this published article (and its Supplementary Information files).

## Electronic supplementary material

supplementary information
