# Supplementary material for: Amniotic membrane promotes focal adhesion remodeling to stimulate cell migration
Source: Sci Rep. 2017 Nov 10;7:15262. doi: 10.1038/s41598-017-15509-z (PMC5681678; doi:10.1038/s41598-017-15509-z)
Supplement: Supplementary file 1 — supplementary information [file 41598_2017_15509_MOESM1_ESM.pdf]

# Amniotic membrane promotes focal adhesion remodeling to stimulate cell migration

Ángel Bernabé-García[1], Sergio Liarte[1], José M. Moraleda[2], Gregorio Castellanos[3] and Francisco J. Nicolás[1,*]

[1]Laboratorio de Oncología Molecular y TGF-ß. IMIB-Arrixaca, El Palmar, Murcia, Spain.

[2]Unidad de Trasplante y Terapia Celular. Servicio Hematología. Hospital Universitario Virgen de la Arrixaca. Universidad de Murcia, Murcia, Spain.

[3]Servicio de Cirugía, Hospital Universitario Virgen de la Arrixaca, El Palmar, Murcia, Spain.

[*]Corresponding author

E-mail: franciscoj.nicolas2@carm.es

Running title: AM stimulates remodeling of focal adhesions.

**Key words**

Amniotic membrane, Cell Migration, Wound healing, Focal Adhesion, Focal Complex, Keratinocyte.

Supplemental figure 1

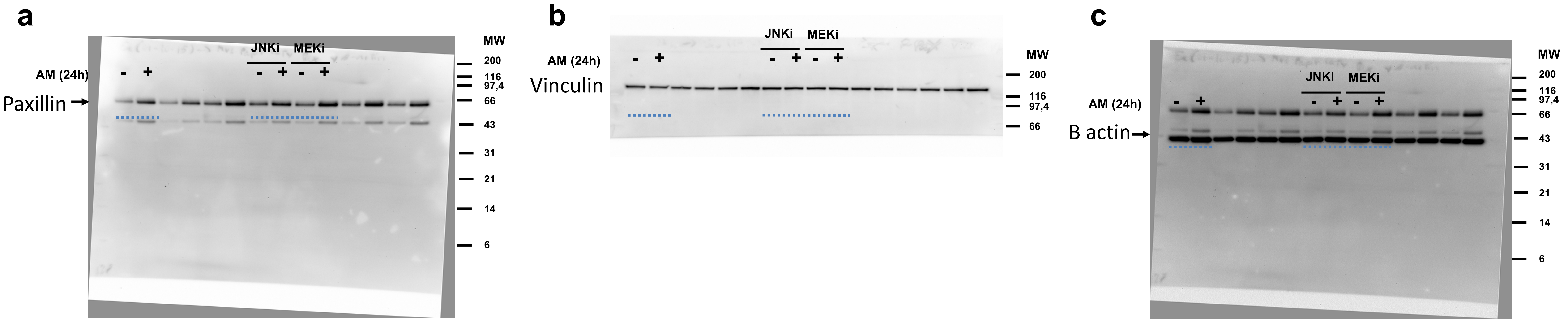


Supp. Fig 1. Full-length blots corresponding to crops showed in Fig 1b. (a) Paxillin. (b) Vinculin. (c) Beta-actin loading. Dashed blue line indicate the portion of the blot that was used in the figure.

# Supplemental figure 2

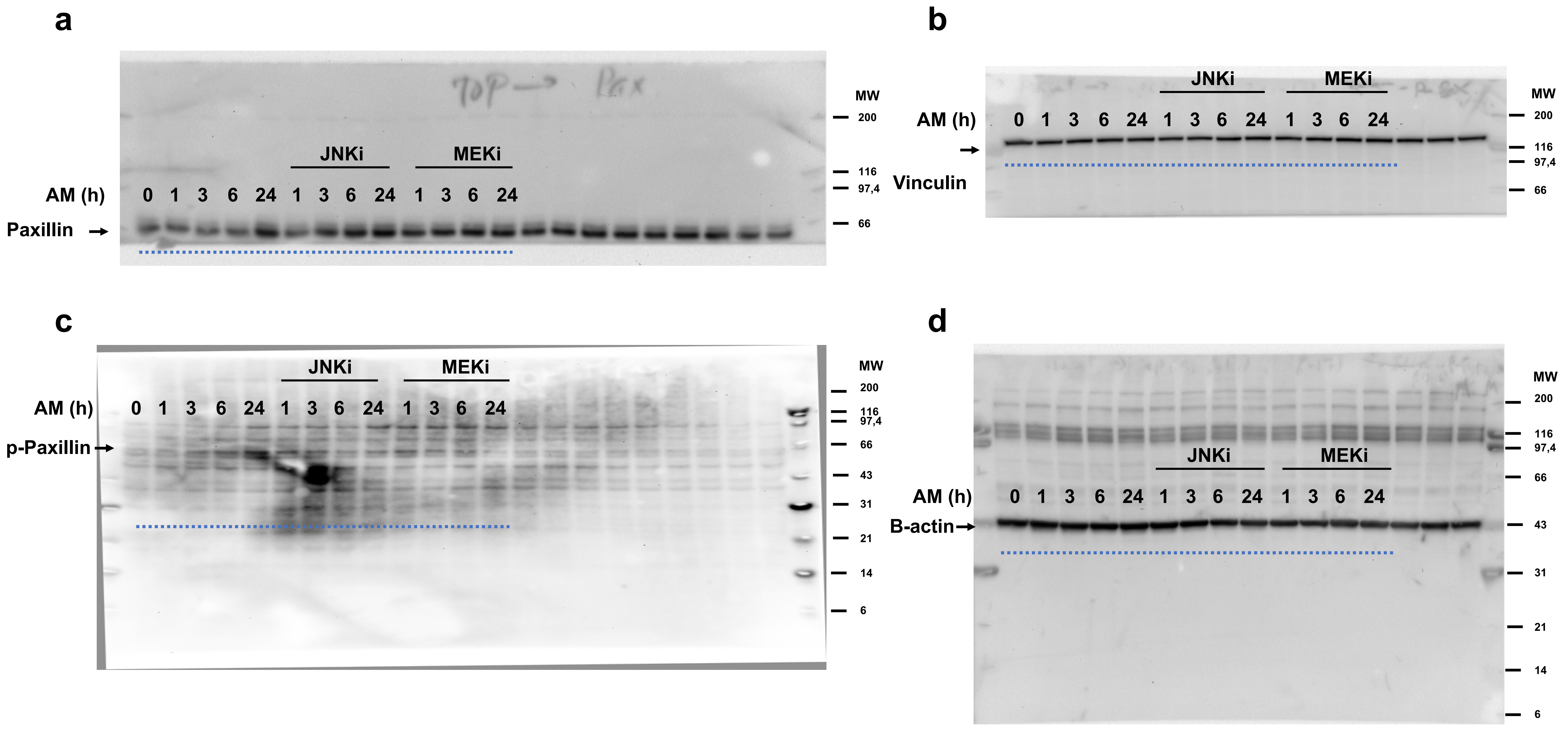


**Supp. Fig 2.** Full-length blots corresponding to crops showed in Fig 3b. (a) Paxillin. (b) Vinculin. (c) Ser 178 Phosphorilated-Paxillin. (d) Beta-actin loading. Dashed blue line indicate the portion of the blot that was used in the figure.

Supplemental figure 3

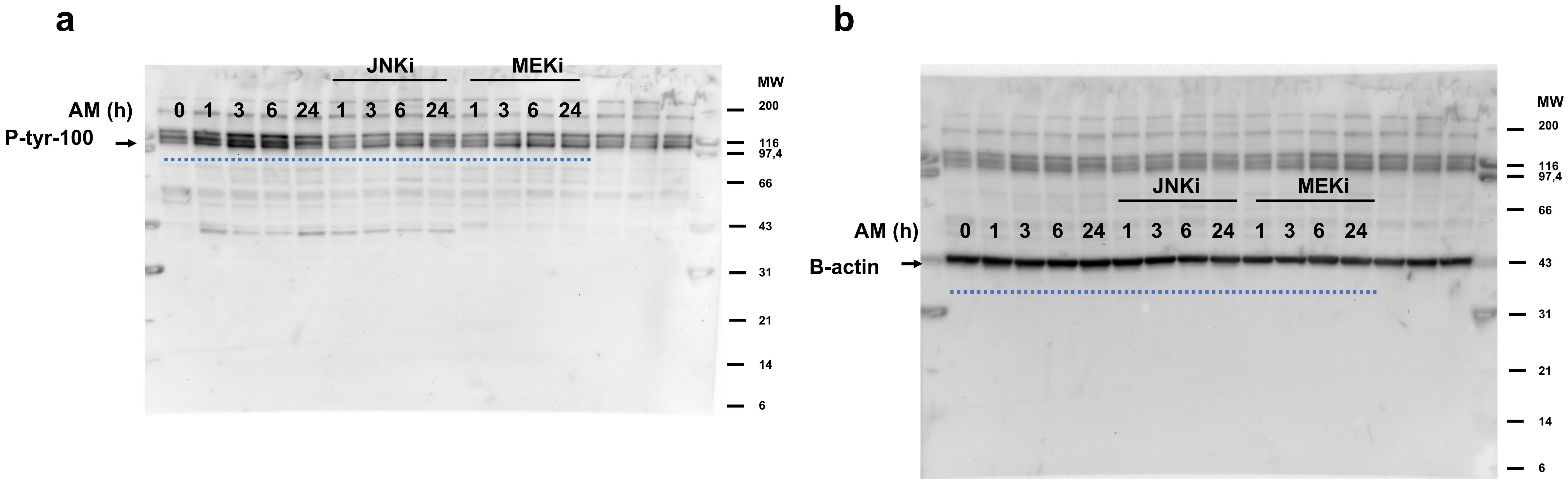


**Supp. Fig 3.** Full-length blots corresponding to crops showed in Fig 5b. (a) P-tyr-100. (b) Beta-actin loading. Dashed blue line indicate the portion of the blot that was used in the figure.
